# Supplementary material for: An evaluation of Mp1p antigen screening for talaromycosis in HIV-infected antiretroviral therapy-naïve population in Guangdong, China
Source: PLoS Negl Trop Dis. 2023 Nov 27;17(11):e0011785. doi: 10.1371/journal.pntd.0011785 (PMC10703259; doi:10.1371/journal.pntd.0011785)
Supplement: S1 Table — (PDF) [file pntd.0011785.s001.pdf]

No.	Result of culture	Result of Mp1p antigen test	Age	Gender	Source of patients	Route of HIV infection	Stage of HIV/AIDS	OIs infection	HBV infection	HCV infection	CD4+ count	CD4+/CD8+ ratio
1	negative	negative	32	male	inpatient	sex	III	positive	negative	negative	1	0.02
2	negative	negative	32	female	inpatient	sex	III	positive	negative	positive	1	0.02
3	positive	positive	43	male	inpatient	sex	IV	positive	negative	negative	1	0.04
4	negative	negative	43	male	inpatient	sex	IV	positive	negative	negative	1	0.00
5	positive	positive	48	male	inpatient	unknown	IV	positive	negative	negative	1	0.01
6	negative	negative	50	male	inpatient	unknown	III	positive	negative	negative	1	0.01
7	positive	positive	51	male	inpatient	sex	IV	positive	negative	negative	1	0.02
8	negative	negative	24	male	inpatient	sex	III	positive	negative	negative	2	0.01
9	negative	negative	43	male	inpatient	sex	III	positive	negative	negative	2	0.01
10	negative	negative	45	male	inpatient	sex	III	positive	negative	negative	2	0.06
11	positive	positive	49	male	inpatient	sex	IV	positive	positive	negative	2	0.01
12	negative	negative	57	male	inpatient	unknown	IV	positive	negative	negative	2	0.01
13	negative	negative	62	male	inpatient	sex	III	positive	negative	negative	2	0.13
14	negative	negative	68	male	inpatient	unknown	IV	positive	negative	negative	2	0.05
15	positive	positive	22	male	inpatient	sex	IV	positive	negative	negative	3	0.01
16	negative	negative	42	male	inpatient	sex	IV	positive	negative	negative	3	0.01
17	positive	positive	47	female	inpatient	sex	IV	positive	negative	negative	3	0.03
18	positive	positive	54	male	inpatient	sex	IV	positive	negative	negative	3	0.06
19	negative	negative	57	male	inpatient	sex	IV	positive	negative	negative	3	0.00
20	positive	positive	25	male	inpatient	sex	IV	positive	negative	negative	4	0.01
21	positive	positive	36	male	inpatient	sex	IV	positive	negative	negative	4	0.01
22	positive	positive	36	male	inpatient	others	IV	positive	negative	negative	4	0.01
23	positive	positive	36	male	inpatient	sex	IV	positive	negative	negative	4	0.03
24	positive	positive	52	male	inpatient	sex	IV	positive	positive	negative	4	0.02
25	positive	negative	53	male	inpatient	others	IV	positive	negative	negative	4	0.05
26	positive	negative	57	female	inpatient	unknown	IV	positive	negative	negative	4	0.03
27	negative	negative	58	male	inpatient	sex	IV	positive	positive	negative	4	0.03
28	positive	positive	66	male	inpatient	unknown	IV	positive	negative	negative	4	0.03
29	positive	positive	69	female	inpatient	sex	IV	positive	negative	negative	4	0.04
30	negative	negative	24	male	inpatient	unknown	III	positive	negative	negative	5	0.01
31	positive	positive	24	male	inpatient	sex	IV	positive	negative	negative	5	0.02

Guangzhou Eighth People's Hospital
Guangzhou Medical University

32	negative	negative	31	male	inpatient	sex	IV	positive	negative	negative	5	0.01
33	positive	positive	40	male	inpatient	unknown	IV	positive	positive	negative	5	0.05
34	negative	negative	42	female	inpatient	unknown	IV	positive	negative	negative	5	0.05
35	positive	positive	46	female	inpatient	blood	IV	positive	negative	negative	5	0.07
36	positive	positive	49	male	inpatient	sex	IV	positive	positive	negative	5	0.02
37	negative	negative	51	male	inpatient	sex	I/II	negative	negative	negative	5	0.00
38	positive	positive	52	male	inpatient	others	IV	positive	positive	negative	5	0.10
39	positive	positive	22	male	inpatient	sex	IV	positive	negative	negative	6	0.03
40	positive	positive	24	male	inpatient	sex	IV	positive	negative	negative	6	0.06
41	positive	positive	25	male	inpatient	sex	IV	positive	negative	negative	6	0.02
42	positive	positive	27	male	inpatient	sex	IV	positive	negative	negative	6	0.02
43	positive	positive	29	male	inpatient	sex	IV	positive	negative	negative	6	0.02
44	negative	negative	34	male	inpatient	sex	III	positive	negative	negative	6	0.01
45	positive	positive	39	male	inpatient	sex	IV	positive	negative	negative	6	0.01
46	positive	positive	40	male	inpatient	sex	IV	positive	positive	negative	6	0.04
47	positive	positive	44	male	inpatient	sex	IV	positive	negative	negative	6	0.09
48	negative	negative	46	male	inpatient	sex	III	positive	negative	negative	6	0.03
49	negative	negative	47	male	inpatient	unknown	III	positive	positive	negative	6	0.03
50	negative	negative	53	male	inpatient	others	IV	positive	negative	negative	6	0.08
51	positive	positive	21	male	inpatient	sex	IV	positive	negative	negative	7	0.06
52	negative	negative	24	female	inpatient	sex	III	positive	negative	negative	7	0.02
53	positive	positive	24	male	inpatient	sex	IV	positive	negative	negative	7	0.03
54	positive	positive	26	male	inpatient	sex	IV	positive	negative	negative	7	0.02
55	negative	positive	34	female	inpatient	unknown	IV	positive	negative	negative	7	0.03
56	positive	positive	42	male	inpatient	sex	IV	positive	negative	negative	7	0.03
57	positive	positive	44	male	inpatient	sex	IV	positive	negative	negative	7	0.04
58	negative	positive	45	male	inpatient	others	IV	positive	negative	negative	7	0.02
59	negative	negative	47	male	inpatient	others	III	positive	negative	negative	7	0.03
60	positive	positive	48	male	inpatient	sex	IV	positive	negative	negative	7	0.04
61	negative	negative	62	male	inpatient	unknown	III	positive	negative	negative	7	0.03
62	negative	negative	71	male	inpatient	others	III	positive	negative	negative	7	0.03
63	negative	negative	30	male	inpatient	sex	III	positive	negative	negative	8	0.02
64	positive	positive	32	male	inpatient	sex	IV	positive	negative	negative	8	0.03
65	positive	positive	35	male	inpatient	others	IV	positive	negative	negative	8	0.04
66	positive	positive	38	male	inpatient	sex	IV	positive	negative	negative	8	0.02

Guangzhou Eighth People's Hospital
Guangzhou Medical University

67	negative	negative	44	male	inpatient	unknown	III	positive	negative	negative	8	0.03
68	negative	negative	54	female	inpatient	sex	IV	positive	negative	negative	8	0.03
69	positive	negative	75	male	inpatient	unknown	IV	positive	negative	negative	8	0.09
70	positive	negative	26	male	inpatient	sex	IV	positive	negative	negative	9	0.03
71	negative	negative	28	male	inpatient	sex	III	positive	negative	negative	9	0.03
72	positive	positive	29	male	inpatient	sex	IV	positive	negative	negative	9	0.10
73	positive	positive	34	male	inpatient	sex	IV	positive	negative	negative	9	0.24
74	positive	positive	38	male	inpatient	sex	IV	positive	positive	negative	9	0.04
75	positive	negative	44	male	inpatient	sex	IV	positive	negative	negative	9	0.08
76	positive	negative	45	male	inpatient	sex	IV	positive	negative	negative	9	0.07
77	negative	negative	47	male	inpatient	sex	IV	positive	negative	negative	9	0.03
78	negative	negative	50	male	inpatient	sex	IV	positive	negative	negative	9	0.04
79	negative	negative	24	male	inpatient	others	IV	positive	negative	negative	10	0.02
80	negative	negative	25	male	inpatient	unknown	III	positive	negative	negative	10	0.04
81	negative	negative	44	male	inpatient	unknown	IV	positive	negative	negative	10	0.03
82	negative	negative	45	male	inpatient	sex	III	positive	negative	negative	10	0.10
83	negative	negative	52	male	inpatient	sex	IV	positive	negative	negative	10	0.07
84	positive	negative	41	male	inpatient	sex	IV	positive	negative	negative	11	0.08
85	negative	negative	50	male	inpatient	sex	IV	positive	negative	negative	11	0.02
86	positive	negative	20	male	inpatient	sex	IV	positive	negative	negative	12	0.03
87	positive	negative	31	male	inpatient	sex	IV	positive	negative	negative	12	0.06
88	negative	negative	33	male	inpatient	sex	III	positive	negative	negative	12	0.01
89	positive	positive	42	female	inpatient	sex	IV	positive	negative	negative	12	0.09
90	negative	negative	46	male	inpatient	unknown	IV	positive	negative	negative	12	0.05
91	positive	positive	46	male	inpatient	sex	IV	positive	positive	negative	12	0.06
92	negative	negative	54	male	inpatient	unknown	III	positive	negative	negative	12	0.06
93	positive	positive	61	male	inpatient	unknown	IV	positive	negative	negative	12	0.10
94	positive	positive	24	male	inpatient	sex	IV	positive	negative	negative	13	0.05
95	positive	positive	46	male	inpatient	sex	IV	positive	negative	negative	13	0.04
96	positive	positive	46	female	inpatient	unknown	IV	positive	positive	negative	13	0.11
97	negative	negative	52	male	inpatient	sex	IV	positive	negative	negative	13	0.08
98	negative	negative	24	male	inpatient	sex	IV	positive	negative	negative	14	0.04
99	positive	positive	36	male	inpatient	sex	IV	positive	negative	negative	14	0.05
100	negative	negative	55	male	inpatient	sex	III	positive	negative	negative	14	0.15
101	positive	positive	25	female	inpatient	sex	IV	positive	negative	negative	15	0.02

Guangzhou Eighth People's Hospital
Guangzhou Medical University

102	positive	positive	31	male	inpatient	unknown	IV	positive	negative	negative	15	0.03
103	positive	negative	38	male	inpatient	others	IV	positive	negative	negative	15	0.03
104	negative	negative	43	male	inpatient	blood	IV	positive	positive	negative	15	0.12
105	negative	negative	74	male	inpatient	sex	III	positive	negative	negative	15	0.05
106	positive	positive	38	female	inpatient	others	IV	positive	negative	negative	16	0.05
107	negative	negative	52	male	inpatient	sex	III	positive	negative	negative	16	0.05
108	positive	negative	52	male	inpatient	sex	IV	positive	negative	negative	16	0.09
109	negative	negative	68	male	inpatient	sex	III	positive	negative	negative	16	0.03
110	positive	positive	23	male	inpatient	others	IV	positive	negative	negative	17	0.07
111	negative	negative	33	male	inpatient	sex	III	positive	negative	negative	17	0.07
112	negative	negative	34	male	inpatient	sex	III	positive	negative	negative	17	0.09
113	negative	negative	40	male	inpatient	sex	III	positive	negative	negative	17	0.03
114	negative	negative	43	male	inpatient	sex	IV	positive	negative	negative	17	0.18
115	positive	negative	64	male	inpatient	unknown	IV	positive	negative	negative	17	0.07
116	negative	negative	25	male	inpatient	sex	IV	positive	negative	negative	18	0.08
117	negative	negative	33	male	inpatient	sex	III	positive	negative	negative	18	0.03
118	negative	negative	37	male	inpatient	unknown	IV	positive	negative	negative	19	0.02
119	negative	negative	52	male	inpatient	sex	III	positive	negative	negative	19	0.06
120	positive	positive	54	male	inpatient	sex	IV	positive	negative	negative	19	0.12
121	negative	negative	21	male	inpatient	sex	III	positive	negative	negative	20	0.05
122	negative	negative	30	male	inpatient	sex	IV	positive	negative	negative	20	0.02
123	negative	negative	36	male	inpatient	sex	IV	positive	negative	negative	20	0.12
124	negative	negative	41	male	inpatient	sex	III	positive	negative	negative	20	0.06
125	negative	negative	44	male	inpatient	sex	III	positive	negative	negative	20	0.06
126	negative	negative	49	female	inpatient	sex	III	positive	negative	negative	20	0.05
127	negative	negative	21	male	inpatient	blood	IV	positive	negative	negative	21	0.05
128	positive	positive	40	male	inpatient	sex	IV	positive	negative	negative	21	0.11
129	negative	negative	44	female	inpatient	sex	III	positive	negative	negative	21	0.08
130	negative	negative	44	female	inpatient	blood	III	positive	negative	negative	21	0.02
131	negative	negative	26	male	inpatient	sex	IV	positive	negative	negative	22	0.09
132	negative	negative	33	female	inpatient	sex	III	positive	negative	negative	22	0.05
133	positive	negative	41	male	inpatient	sex	IV	positive	negative	negative	22	0.06
134	negative	negative	71	male	inpatient	blood	III	positive	negative	negative	22	0.11
135	negative	negative	30	male	inpatient	sex	III	positive	negative	negative	23	0.07
136	negative	negative	48	male	inpatient	sex	III	positive	positive	negative	23	0.09

Guangzhou Eighth People's Hospital
Guangzhou Medical University

137	negative	negative	59	female	inpatient	sex	III	positive	negative	negative	23	0.33
138	negative	negative	65	male	inpatient	sex	I/II	positive	negative	negative	23	0.13
139	positive	positive	74	male	inpatient	unknown	IV	positive	negative	negative	23	0.03
140	positive	positive	36	male	inpatient	sex	IV	positive	negative	negative	24	0.06
141	negative	negative	59	male	inpatient	unknown	IV	positive	negative	negative	24	0.09
142	negative	negative	29	male	inpatient	others	III	positive	negative	negative	25	0.09
143	positive	positive	31	male	inpatient	sex	IV	positive	negative	negative	25	0.09
144	negative	negative	35	male	inpatient	unknown	III	positive	negative	negative	25	0.05
145	positive	positive	35	male	inpatient	sex	IV	positive	negative	negative	25	0.07
146	negative	negative	46	female	inpatient	sex	III	positive	negative	negative	25	0.10
147	negative	negative	56	male	inpatient	sex	III	positive	negative	negative	25	0.11
148	negative	negative	61	male	inpatient	unknown	III	positive	negative	negative	25	0.06
149	negative	negative	40	male	inpatient	others	IV	positive	negative	negative	26	0.07
150	negative	negative	43	male	inpatient	sex	III	positive	negative	negative	26	0.11
151	positive	negative	52	male	inpatient	sex	IV	positive	negative	positive	27	0.06
152	positive	negative	56	male	inpatient	others	IV	positive	negative	negative	27	0.05
153	positive	negative	42	male	inpatient	sex	IV	positive	negative	positive	28	0.10
154	negative	negative	48	male	inpatient	sex	III	positive	negative	negative	28	0.04
155	negative	negative	49	male	inpatient	unknown	III	positive	negative	negative	28	0.19
156	negative	negative	58	female	inpatient	sex	IV	positive	negative	negative	28	0.05
157	positive	positive	30	male	inpatient	sex	IV	positive	negative	negative	29	0.10
158	negative	negative	50	male	inpatient	sex	III	positive	negative	negative	29	0.04
159	negative	negative	64	female	inpatient	blood	IV	positive	positive	positive	30	0.20
160	negative	negative	41	male	inpatient	sex	III	positive	positive	negative	31	0.16
161	positive	negative	72	male	inpatient	sex	IV	positive	positive	negative	31	0.07
162	negative	negative	22	male	inpatient	sex	IV	positive	positive	negative	32	0.07
163	positive	negative	38	male	inpatient	sex	IV	positive	negative	negative	32	0.17
164	negative	negative	26	male	inpatient	sex	III	positive	positive	negative	33	0.07
165	negative	negative	40	male	inpatient	sex	III	positive	positive	negative	33	0.15
166	negative	negative	46	male	inpatient	blood	III	positive	negative	positive	33	0.03
167	negative	negative	53	male	inpatient	unknown	III	positive	negative	negative	33	0.41
168	negative	negative	30	female	inpatient	sex	III	positive	negative	negative	34	0.06
169	negative	negative	46	male	inpatient	unknown	III	positive	negative	negative	34	0.06
170	positive	negative	51	male	inpatient	others	IV	positive	negative	negative	35	0.28
171	positive	positive	23	male	inpatient	sex	IV	positive	negative	negative	37	0.14

Guangzhou Eighth People's Hospital
Guangzhou Medical University

172	negative	negative	61	male	inpatient	others	III	positive	negative	negative	37	0.16
173	positive	positive	31	male	inpatient	sex	IV	positive	negative	negative	38	0.09
174	negative	negative	66	male	inpatient	sex	IV	positive	negative	negative	38	0.17
175	positive	positive	32	male	inpatient	others	IV	positive	positive	negative	39	0.07
176	negative	positive	34	male	inpatient	sex	III	positive	negative	negative	39	0.09
177	negative	negative	44	male	inpatient	blood	III	positive	negative	negative	39	0.09
178	positive	negative	35	male	inpatient	others	IV	positive	negative	negative	40	0.07
179	negative	negative	33	male	inpatient	sex	III	positive	negative	positive	41	0.17
180	negative	positive	41	male	inpatient	sex	III	positive	negative	negative	41	0.03
181	negative	negative	54	male	inpatient	sex	III	positive	negative	negative	41	0.05
182	positive	negative	35	male	inpatient	sex	IV	positive	positive	negative	42	0.16
183	negative	negative	40	female	inpatient	sex	III	positive	negative	negative	42	0.09
184	negative	negative	45	male	inpatient	sex	III	positive	positive	negative	42	0.13
185	negative	negative	46	male	inpatient	sex	III	positive	negative	negative	42	0.20
186	negative	negative	71	female	inpatient	unknown	III	positive	negative	negative	42	0.14
187	negative	negative	26	male	inpatient	sex	III	positive	negative	negative	43	0.06
188	positive	positive	27	male	inpatient	sex	IV	positive	negative	negative	44	0.18
189	negative	negative	36	male	inpatient	sex	III	positive	negative	negative	44	0.14
190	negative	negative	41	male	inpatient	others	III	positive	positive	negative	44	0.15
191	positive	positive	42	male	inpatient	others	IV	positive	positive	negative	44	0.11
192	negative	negative	55	female	inpatient	blood	IV	positive	negative	positive	44	0.22
193	negative	negative	65	male	inpatient	sex	III	positive	negative	negative	44	0.07
194	positive	positive	23	male	inpatient	sex	IV	positive	negative	negative	45	0.05
195	positive	negative	47	female	inpatient	sex	IV	positive	negative	positive	48	0.15
196	negative	negative	54	female	inpatient	others	IV	positive	negative	negative	48	0.15
197	negative	negative	33	male	inpatient	sex	III	positive	negative	negative	49	0.20
198	positive	negative	33	female	inpatient	sex	IV	positive	negative	negative	50	0.10
199	positive	positive	38	male	inpatient	unknown	IV	positive	negative	negative	51	0.12
200	negative	negative	50	male	inpatient	sex	III	positive	negative	negative	51	0.09
201	positive	positive	52	male	inpatient	sex	IV	positive	negative	negative	51	0.31
202	negative	negative	77	male	inpatient	others	IV	positive	negative	negative	51	0.45
203	negative	negative	32	male	inpatient	others	III	positive	negative	negative	52	0.11
204	negative	negative	47	male	inpatient	unknown	III	positive	negative	negative	52	0.10
205	positive	negative	47	male	inpatient	sex	IV	positive	negative	negative	54	0.08
206	negative	negative	42	female	inpatient	others	III	positive	negative	negative	55	0.10

Guangzhou Eighth People's Hospital
Guangzhou Medical University

207	negative	negative	70	male	inpatient	sex	III	positive	positive	negative	57	0.15
208	negative	negative	70	male	inpatient	sex	III	positive	negative	negative	58	0.18
209	negative	negative	66	male	inpatient	sex	III	positive	positive	negative	59	0.44
210	negative	negative	53	male	inpatient	unknown	III	positive	negative	negative	60	0.12
211	negative	negative	42	male	inpatient	blood	III	positive	negative	positive	61	0.10
212	negative	negative	41	male	inpatient	sex	III	positive	negative	negative	63	0.16
213	negative	negative	61	male	inpatient	sex	IV	positive	negative	negative	63	0.08
214	positive	positive	30	male	inpatient	sex	IV	positive	negative	negative	64	0.10
215	negative	negative	58	male	inpatient	unknown	I/II	negative	positive	negative	64	0.38
216	negative	negative	60	female	inpatient	sex	III	positive	negative	negative	64	0.17
217	negative	negative	37	male	inpatient	sex	IV	positive	negative	negative	65	0.15
218	negative	negative	49	male	inpatient	sex	IV	positive	negative	negative	66	0.08
219	negative	negative	45	male	inpatient	sex	III	positive	negative	negative	67	0.13
220	negative	negative	57	male	inpatient	sex	IV	positive	negative	negative	68	0.10
221	negative	negative	46	female	inpatient	unknown	III	positive	negative	negative	69	0.27
222	negative	negative	55	male	inpatient	sex	III	positive	negative	negative	69	0.12
223	negative	negative	63	male	inpatient	sex	III	positive	negative	negative	69	0.13
224	negative	negative	68	female	inpatient	sex	I/II	negative	negative	negative	70	0.22
225	negative	negative	35	male	inpatient	unknown	III	positive	negative	negative	71	0.25
226	positive	positive	49	female	inpatient	sex	IV	positive	positive	negative	72	0.11
227	negative	negative	67	male	inpatient	others	III	positive	negative	negative	72	0.08
228	negative	negative	74	male	inpatient	unknown	III	positive	negative	negative	72	0.19
229	negative	negative	65	male	inpatient	sex	III	positive	negative	negative	73	0.11
230	positive	negative	27	male	inpatient	others	IV	positive	negative	negative	74	0.13
231	negative	negative	35	male	inpatient	unknown	III	positive	negative	negative	74	0.25
232	negative	negative	30	male	inpatient	sex	I/II	negative	negative	negative	75	0.13
233	negative	negative	46	male	inpatient	sex	IV	positive	negative	negative	75	0.06
234	positive	negative	44	female	inpatient	unknown	IV	positive	negative	negative	76	0.31
235	negative	negative	25	male	inpatient	sex	I/II	negative	negative	negative	79	0.07
236	negative	positive	56	male	inpatient	sex	III	positive	negative	negative	80	0.17
237	negative	negative	50	male	inpatient	unknown	I/II	negative	positive	positive	87	0.69
238	negative	negative	53	male	inpatient	sex	III	positive	negative	negative	87	0.10
239	negative	negative	56	male	inpatient	sex	IV	positive	positive	negative	87	0.15
240	negative	negative	46	male	inpatient	sex	III	positive	negative	negative	89	0.14
241	negative	negative	58	male	inpatient	sex	III	positive	negative	negative	92	0.23

Guangzhou Eighth People's Hospital
Guangzhou Medical University

242	negative	negative	67	male	inpatient	others	III	positive	negative	negative	92	0.06
243	negative	negative	40	male	inpatient	sex	IV	positive	negative	negative	93	0.06
244	negative	negative	54	male	inpatient	unknown	III	positive	negative	negative	93	0.45
245	negative	negative	38	female	inpatient	sex	I/II	negative	negative	negative	94	0.30
246	negative	negative	41	male	inpatient	unknown	III	positive	negative	negative	95	0.12
247	negative	negative	29	male	inpatient	sex	I/II	negative	negative	negative	96	0.22
248	negative	negative	31	male	inpatient	sex	III	positive	negative	positive	97	0.15
249	negative	negative	45	male	inpatient	sex	III	positive	positive	negative	50	0.15
250	negative	negative	54	male	inpatient	sex	III	positive	negative	negative	100	0.13
251	negative	negative	38	male	inpatient	others	IV	positive	positive	negative	101	0.06
252	negative	negative	62	male	inpatient	unknown	III	positive	negative	negative	106	0.24
253	negative	negative	31	male	inpatient	sex	IV	positive	negative	negative	108	0.06
254	negative	negative	45	female	inpatient	sex	III	positive	negative	negative	116	0.21
255	negative	negative	51	male	inpatient	unknown	III	positive	negative	negative	116	0.19
256	negative	negative	67	male	inpatient	sex	III	positive	positive	negative	116	0.17
257	negative	negative	47	male	inpatient	sex	III	positive	negative	negative	119	0.45
258	negative	negative	22	male	inpatient	sex	III	positive	negative	negative	120	0.22
259	negative	negative	36	male	inpatient	sex	IV	positive	negative	negative	121	0.18
260	negative	negative	48	male	inpatient	sex	III	positive	positive	negative	124	0.25
261	negative	negative	33	male	inpatient	unknown	III	positive	negative	negative	129	0.28
262	negative	negative	24	male	inpatient	sex	III	positive	negative	negative	130	1.05
263	negative	negative	42	male	inpatient	blood	III	positive	negative	positive	132	0.21
264	negative	negative	46	male	inpatient	sex	III	positive	negative	negative	133	0.48
265	negative	positive	40	male	inpatient	sex	III	positive	positive	negative	134	0.27
266	negative	negative	43	female	inpatient	blood	III	positive	negative	positive	138	0.61
267	negative	negative	57	female	inpatient	sex	III	positive	negative	negative	138	0.16
268	negative	negative	51	male	inpatient	sex	III	positive	negative	negative	139	0.22
269	negative	negative	60	male	inpatient	unknown	III	positive	negative	negative	144	0.34
270	negative	negative	28	male	inpatient	unknown	III	positive	negative	negative	147	0.50
271	negative	negative	31	male	inpatient	sex	III	positive	negative	negative	156	0.36
272	negative	negative	63	male	inpatient	sex	IV	positive	negative	negative	161	0.63
273	negative	negative	33	male	inpatient	others	IV	positive	negative	negative	83	0.05
274	negative	negative	58	male	inpatient	sex	III	positive	negative	negative	165	0.31
275	negative	negative	75	male	inpatient	sex	I/II	negative	negative	negative	165	0.31
276	negative	negative	70	male	inpatient	sex	III	positive	negative	negative	168	0.46

Guangzhou Eighth People's Hospital
Guangzhou Medical University

277	negative	negative	36	female	inpatient	sex	I/II	negative	negative	negative	170	0.27
278	negative	negative	32	male	inpatient	sex	III	positive	negative	negative	172	0.13
279	negative	negative	19	male	inpatient	others	IV	positive	negative	negative	179	0.10
280	negative	negative	54	female	inpatient	others	III	positive	positive	negative	179	0.22
281	negative	negative	27	male	inpatient	sex	I/II	negative	negative	negative	181	0.17
282	negative	negative	38	male	inpatient	sex	III	positive	negative	negative	189	0.20
283	negative	positive	33	male	inpatient	blood	III	positive	positive	positive	190	0.12
284	negative	negative	48	male	inpatient	blood	I/II	negative	positive	positive	190	0.22
285	negative	negative	45	male	inpatient	others	I/II	negative	positive	negative	192	0.34
286	negative	negative	75	male	inpatient	unknown	III	positive	negative	negative	193	0.09
287	negative	negative	49	male	inpatient	others	III	positive	negative	negative	87	0.14
288	negative	negative	50	male	inpatient	others	I/II	negative	negative	negative	202	0.26
289	negative	negative	53	male	inpatient	sex	III	positive	negative	negative	204	0.19
290	positive	negative	58	male	inpatient	sex	IV	positive	negative	negative	207	0.04
291	negative	negative	42	female	inpatient	sex	III	positive	negative	negative	210	0.39
292	negative	negative	49	female	inpatient	sex	III	positive	positive	negative	216	0.19
293	negative	negative	79	male	inpatient	sex	III	positive	negative	negative	216	0.18
294	negative	negative	48	male	inpatient	others	III	positive	negative	negative	223	0.22
295	negative	negative	61	male	inpatient	unknown	III	positive	negative	negative	223	0.06
296	negative	negative	41	male	inpatient	sex	III	positive	positive	negative	224	0.55
297	negative	negative	61	female	inpatient	unknown	I/II	negative	negative	negative	231	0.25
298	negative	negative	70	male	inpatient	sex	III	positive	negative	negative	233	0.24
299	negative	negative	40	male	inpatient	unknown	I/II	negative	negative	negative	243	0.48
300	negative	negative	45	male	inpatient	blood	III	positive	negative	positive	249	0.23
301	negative	negative	72	male	inpatient	others	III	positive	negative	negative	249	0.10
302	negative	negative	73	male	inpatient	unknown	I/II	negative	negative	negative	254	0.15
303	negative	negative	24	male	inpatient	sex	I/II	negative	negative	negative	257	0.20
304	negative	negative	39	male	inpatient	sex	III	positive	negative	negative	258	0.33
305	negative	negative	45	male	inpatient	sex	III	positive	negative	positive	260	0.15
306	negative	negative	22	male	inpatient	unknown	III	positive	negative	negative	266	0.13
307	negative	negative	39	male	inpatient	blood	III	positive	negative	positive	266	0.69
308	negative	negative	45	male	inpatient	sex	III	positive	negative	positive	266	0.51
309	negative	negative	60	male	inpatient	unknown	III	positive	negative	negative	271	0.73
310	negative	negative	44	male	inpatient	sex	I/II	positive	negative	negative	279	0.26
311	negative	negative	74	male	inpatient	unknown	III	positive	negative	negative	284	0.26

Guangzhou Eighth People's Hospital
Guangzhou Medical University

312	negative	negative	44	male	inpatient	sex	I/II	negative	negative	negative	298	0.38
313	negative	negative	52	female	inpatient	others	I/II	negative	negative	negative	302	0.58
314	negative	negative	23	male	inpatient	sex	I/II	negative	negative	negative	309	0.83
315	negative	negative	22	male	inpatient	sex	I/II	negative	negative	negative	321	0.40
316	negative	negative	23	male	inpatient	sex	III	positive	positive	negative	325	0.29
317	negative	negative	55	male	inpatient	others	I/II	positive	negative	negative	330	0.33
318	negative	negative	41	female	inpatient	unknown	I/II	negative	positive	negative	331	0.10
319	negative	negative	35	male	inpatient	blood	I/II	positive	negative	positive	354	0.18
320	negative	negative	44	male	inpatient	unknown	III	positive	negative	negative	355	0.36
321	negative	negative	25	male	inpatient	sex	I/II	negative	negative	negative	364	0.41
322	negative	negative	45	male	inpatient	blood	I/II	negative	negative	positive	372	0.73
323	negative	negative	53	male	inpatient	sex	I/II	negative	negative	negative	376	0.29
324	negative	negative	73	male	inpatient	sex	III	positive	negative	negative	381	0.36
325	negative	negative	27	male	inpatient	unknown	I/II	negative	negative	negative	384	0.47
326	negative	negative	65	female	inpatient	sex	III	positive	negative	negative	389	0.59
327	negative	negative	63	male	inpatient	sex	III	positive	negative	negative	391	0.24
328	negative	negative	62	female	inpatient	sex	I/II	positive	negative	negative	393	0.41
329	negative	negative	74	male	inpatient	unknown	I/II	negative	positive	negative	398	0.69
330	negative	negative	41	male	inpatient	blood	I/II	negative	negative	positive	405	0.47
331	negative	negative	36	male	inpatient	others	I/II	negative	negative	negative	407	0.45
332	negative	negative	23	male	inpatient	sex	I/II	negative	negative	negative	412	0.22
333	negative	negative	53	female	inpatient	unknown	I/II	negative	negative	negative	442	0.60
334	negative	negative	60	male	inpatient	sex	I/II	negative	negative	positive	462	1.16
335	negative	negative	62	male	inpatient	sex	III	positive	positive	negative	486	0.38
336	negative	negative	20	male	inpatient	sex	III	positive	negative	negative	508	0.37
337	negative	negative	26	male	inpatient	sex	I/II	negative	negative	negative	528	0.40
338	negative	negative	40	male	inpatient	blood	I/II	negative	negative	positive	544	0.23
339	negative	negative	55	male	inpatient	sex	III	positive	positive	negative	600	0.53
340	negative	negative	42	female	inpatient	blood	I/II	negative	negative	negative	626	0.31
341	negative	negative	37	male	inpatient	sex	I/II	negative	negative	negative	687	1.62
342	negative	negative	64	male	inpatient	sex	III	positive	negative	negative	781	0.33
343	negative	negative	21	male	inpatient	sex	I/II	negative	positive	negative	962	0.76
344	negative	negative	33	female	inpatient	unknown	III	positive	negative	positive	1084	1.91
345	none	negative	42	male	inpatient	sex	I/II	negative	negative	negative	133	0.19
346	negative	negative	35	male	inpatient	sex	III	positive	negative	negative	166	0.31

Guangzhou Eighth People's Hospital
Guangzhou Medical University

347	none	negative	24	male	inpatient	sex	I/II	negative	negative	negative	188	0.29
348	negative	negative	46	male	inpatient	sex	I/II	negative	negative	negative	272	0.16
349	negative	negative	49	male	inpatient	sex	III	positive	positive	negative	276	0.15
350	none	negative	41	female	inpatient	sex	I/II	negative	negative	negative	296	0.66
351	none	negative	35	male	inpatient	sex	I/II	negative	negative	negative	494	0.42
352	none	negative	73	male	inpatient	unknown	I/II	positive	negative	negative	647	0.20
353	negative	negative	51	male	inpatient	sex	IV	positive	negative	negative	14	0.04
354	negative	negative	43	female	inpatient	sex	IV	positive	negative	negative	9	0.04
355	none	negative	42	male	outpatient	sex	I/II	negative	positive	negative	319	0.38
356	none	negative	43	male	outpatient	sex	I/II	negative	positive	negative	158	0.18
357	none	negative	29	female	outpatient	unknown	I/II	negative	negative	negative	694	0.89
358	none	negative	35	male	outpatient	sex	I/II	negative	negative	negative	1	0.00
359	none	negative	43	male	outpatient	blood	I/II	negative	negative	positive	680	0.58
360	none	negative	28	male	outpatient	sex	I/II	negative	negative	negative	146	0.09
361	none	negative	28	male	outpatient	sex	I/II	negative	negative	negative	58	0.07
362	none	negative	35	male	outpatient	sex	I/II	negative	negative	negative	304	0.21
363	none	negative	28	male	outpatient	sex	I/II	negative	negative	negative	744	0.21
364	none	negative	27	male	outpatient	sex	I/II	negative	positive	negative	401	0.55
365	none	negative	27	male	outpatient	sex	I/II	negative	negative	negative	454	0.43
366	none	negative	37	male	outpatient	sex	I/II	negative	negative	negative	323	0.17
367	none	negative	43	male	outpatient	sex	I/II	negative	negative	negative	361	0.39
368	none	negative	31	male	outpatient	sex	I/II	negative	negative	negative	261	0.17
369	none	negative	45	male	outpatient	sex	I/II	negative	negative	negative	85	0.25
370	none	negative	25	male	outpatient	sex	I/II	negative	negative	negative	239	0.35
371	none	negative	34	male	outpatient	sex	I/II	negative	negative	negative	462	0.34
372	none	negative	36	male	outpatient	sex	I/II	negative	negative	negative	126	0.14
373	none	negative	52	male	outpatient	sex	I/II	negative	negative	negative	7	0.06
374	none	negative	23	male	outpatient	sex	I/II	negative	negative	negative	562	0.65
375	none	negative	36	male	outpatient	sex	I/II	negative	negative	negative	15	0.02
376	none	negative	37	male	outpatient	sex	I/II	negative	negative	negative	319	0.39
377	none	negative	40	male	outpatient	sex	I/II	negative	negative	negative	49	0.23
378	none	negative	36	male	outpatient	sex	I/II	negative	negative	negative	185	0.20
379	none	negative	36	male	outpatient	sex	I/II	negative	negative	negative	175	0.34
380	none	negative	27	male	outpatient	unknown	I/II	negative	negative	negative	247	0.25
381	none	negative	27	male	outpatient	sex	I/II	negative	negative	negative	611	0.25

Guangzhou Eighth People's Hospital
Guangzhou Medical University

382	none	negative	24	male	outpatient	sex	I/II	negative	negative	negative	440	0.49
383	none	negative	25	male	outpatient	sex	I/II	negative	negative	negative	257	0.46
384	none	negative	24	male	outpatient	others	I/II	negative	negative	negative	178	0.26
385	none	negative	21	male	outpatient	sex	I/II	negative	negative	negative	179	0.11
386	none	negative	46	male	outpatient	sex	I/II	negative	negative	negative	369	0.42
387	none	negative	32	male	outpatient	sex	I/II	negative	negative	negative	574	0.43
388	none	negative	31	male	outpatient	sex	I/II	negative	negative	negative	443	0.12
389	none	positive	41	male	outpatient	others	I/II	negative	positive	negative	180	0.44
390	none	negative	28	male	outpatient	sex	I/II	negative	positive	negative	178	0.42
391	none	negative	32	male	outpatient	sex	I/II	negative	negative	negative	300	0.40
392	none	negative	38	female	outpatient	unknown	I/II	negative	negative	negative	157	0.15
393	none	negative	31	male	outpatient	sex	I/II	negative	negative	negative	278	0.20
394	none	negative	30	male	outpatient	sex	I/II	negative	negative	negative	941	0.59
395	none	negative	22	male	outpatient	sex	I/II	negative	negative	negative	240	0.32
396	none	negative	27	male	outpatient	sex	I/II	negative	negative	negative	421	0.63
397	none	positive	48	male	outpatient	sex	III	positive	negative	negative	36	0.25
398	none	negative	41	male	outpatient	sex	I/II	negative	positive	negative	77	0.06
399	none	negative	30	male	outpatient	sex	I/II	negative	negative	negative	482	0.42
400	none	positive	23	male	outpatient	sex	I/II	negative	negative	negative	261	0.35
401	none	positive	24	male	outpatient	sex	I/II	positive	negative	negative	178	0.15
402	none	negative	23	male	outpatient	sex	I/II	negative	negative	negative	811	0.47
403	none	negative	50	male	outpatient	sex	I/II	negative	negative	negative	457	0.22
404	none	negative	25	male	outpatient	sex	I/II	negative	negative	negative	263	0.26
405	none	negative	42	male	outpatient	sex	I/II	negative	negative	negative	67	0.04
406	none	negative	24	male	outpatient	sex	I/II	negative	negative	negative	274	0.25
407	none	negative	20	male	outpatient	sex	I/II	negative	negative	negative	267	0.22
408	none	negative	33	male	outpatient	sex	I/II	negative	negative	negative	61	0.12
409	none	negative	69	male	outpatient	sex	I/II	negative	negative	negative	164	0.20
410	none	negative	31	male	outpatient	unknown	I/II	negative	negative	negative	626	0.55
411	none	negative	29	male	outpatient	sex	I/II	negative	negative	negative	796	0.45
412	none	negative	41	female	outpatient	sex	I/II	negative	positive	negative	703	0.55
413	none	negative	23	male	outpatient	sex	I/II	negative	negative	negative	280	0.43
414	none	negative	29	male	outpatient	sex	I/II	negative	negative	negative	268	0.30
415	none	negative	32	male	outpatient	sex	I/II	negative	negative	negative	393	0.36
416	none	negative	20	female	outpatient	sex	I/II	negative	negative	negative	244	0.26

Guangzhou Eighth People's Hospital
Guangzhou Medical University

417	none	negative	27	male	outpatient	sex	I/II	negative	negative	negative	189	0.19
418	none	negative	28	male	outpatient	sex	I/II	negative	negative	negative	263	0.70
419	none	negative	29	male	outpatient	sex	I/II	negative	negative	negative	264	0.21
420	none	negative	22	male	outpatient	sex	I/II	negative	negative	negative	386	0.43
421	none	negative	22	male	outpatient	sex	I/II	negative	negative	negative	338	0.42
422	none	negative	21	male	outpatient	sex	I/II	negative	negative	negative	202	0.21
423	none	negative	35	male	outpatient	sex	I/II	negative	negative	negative	209	0.32
424	none	negative	24	male	outpatient	sex	I/II	negative	negative	negative	324	0.52
425	none	negative	30	male	outpatient	sex	I/II	negative	negative	negative	124	0.24
426	none	negative	21	male	outpatient	sex	I/II	negative	negative	negative	320	0.41
427	none	negative	49	male	outpatient	sex	I/II	negative	negative	negative	346	0.28
428	none	negative	40	female	outpatient	sex	I/II	negative	negative	negative	474	0.27
429	none	negative	36	male	outpatient	sex	I/II	negative	negative	negative	366	0.16
430	none	negative	40	female	outpatient	sex	I/II	negative	negative	negative	401	0.34
431	none	negative	32	male	outpatient	sex	I/II	negative	negative	negative	315	0.57
432	none	negative	34	male	outpatient	sex	I/II	negative	negative	negative	447	0.72
433	none	negative	38	male	outpatient	sex	I/II	negative	negative	negative	311	0.57
434	none	negative	57	male	outpatient	sex	I/II	negative	negative	negative	329	0.67
435	none	negative	24	male	outpatient	sex	I/II	negative	negative	negative	327	0.30
436	none	negative	49	male	outpatient	sex	I/II	negative	negative	negative	634	0.80
437	none	negative	49	male	outpatient	sex	I/II	negative	negative	negative	225	0.07
438	none	negative	33	male	outpatient	sex	I/II	negative	negative	negative	316	0.58
439	none	negative	45	male	outpatient	sex	I/II	negative	negative	negative	101	0.10
440	none	negative	24	male	outpatient	unknown	I/II	negative	negative	negative	251	0.30
441	none	negative	24	male	outpatient	sex	I/II	negative	negative	negative	34	0.07
442	none	negative	21	male	outpatient	sex	I/II	negative	negative	negative	270	0.13
443	none	negative	29	male	outpatient	sex	I/II	negative	negative	negative	230	0.34
444	none	negative	20	male	outpatient	sex	I/II	negative	negative	negative	307	0.56
445	none	negative	53	male	outpatient	sex	I/II	negative	negative	negative	193	0.21
446	none	negative	27	male	outpatient	sex	I/II	negative	negative	negative	475	0.77
447	none	negative	28	male	outpatient	sex	I/II	negative	negative	negative	344	0.24
448	none	negative	27	male	outpatient	sex	I/II	negative	negative	negative	555	0.61
449	none	negative	34	male	outpatient	sex	I/II	negative	negative	negative	307	0.35
450	none	negative	35	male	outpatient	sex	I/II	negative	negative	negative	224	0.17
451	none	negative	34	male	outpatient	sex	I/II	negative	negative	negative	196	0.33

Guangzhou Eighth People's Hospital
Guangzhou Medical University

452	none	negative	28	male	outpatient	sex	I/II	negative	negative	negative	318	0.25
453	none	negative	28	male	outpatient	sex	I/II	negative	negative	negative	334	0.43
454	none	negative	25	male	outpatient	sex	I/II	negative	positive	negative	131	0.28
455	none	negative	32	male	outpatient	sex	I/II	negative	negative	negative	225	0.25
456	none	positive	66	male	outpatient	sex	I/II	negative	negative	negative	321	0.46
457	none	positive	36	male	outpatient	sex	I/II	negative	negative	negative	191	0.13
458	none	negative	34	male	outpatient	sex	I/II	negative	negative	negative	249	0.23
459	none	negative	18	male	outpatient	sex	I/II	negative	negative	negative	271	0.31
460	none	negative	56	male	outpatient	sex	I/II	negative	negative	negative	27	0.09
461	none	positive	37	male	outpatient	others	I/II	negative	negative	negative	13	0.09
462	none	negative	22	male	outpatient	sex	I/II	negative	negative	negative	322	0.13
463	none	negative	75	male	outpatient	others	I/II	negative	negative	negative	199	0.25
464	none	negative	43	male	outpatient	sex	I/II	negative	negative	negative	258	0.30
465	none	negative	29	male	outpatient	sex	I/II	negative	negative	negative	557	0.93
466	none	negative	21	male	outpatient	sex	I/II	negative	negative	negative	328	0.41
467	none	negative	22	male	outpatient	sex	I/II	negative	negative	negative	408	0.51
468	none	negative	30	male	outpatient	sex	I/II	negative	negative	negative	402	0.37
469	none	negative	49	female	outpatient	sex	I/II	negative	negative	negative	365	0.33
470	none	negative	44	male	outpatient	sex	I/II	negative	negative	negative	158	1.30
471	none	negative	34	male	outpatient	sex	I/II	negative	negative	negative	238	0.37
472	none	negative	24	male	outpatient	sex	I/II	negative	negative	negative	320	0.44
473	none	negative	18	male	outpatient	sex	I/II	negative	negative	negative	465	0.27
474	none	negative	20	male	outpatient	sex	I/II	negative	negative	negative	244	0.42
475	none	negative	44	male	outpatient	sex	I/II	negative	negative	negative	354	0.62
476	positive	positive	24	male	outpatient	sex	IV	positive	negative	negative	12	0.03
477	none	negative	39	male	outpatient	sex	I/II	negative	positive	negative	10	0.01
478	none	negative	32	male	outpatient	sex	I/II	negative	negative	negative	133	0.32
479	none	negative	75	male	outpatient	others	I/II	negative	negative	negative	270	0.48
480	none	negative	53	female	outpatient	others	I/II	negative	negative	negative	239	0.40
481	none	negative	28	male	outpatient	sex	I/II	negative	negative	negative	579	1.44
482	none	negative	25	male	outpatient	sex	I/II	negative	negative	negative	309	0.32
483	none	negative	26	male	outpatient	sex	I/II	negative	negative	negative	135	0.10
484	none	positive	46	male	outpatient	sex	I/II	negative	negative	negative	38	0.05
485	none	negative	27	male	outpatient	sex	I/II	negative	negative	negative	250	0.20
486	none	negative	24	male	outpatient	sex	I/II	negative	positive	negative	210	0.32

Guangzhou Eighth People's Hospital
Guangzhou Medical University

487	none	negative	46	male	outpatient	sex	I/II	negative	negative	negative	134	0.42
488	none	negative	42	male	outpatient	sex	I/II	negative	negative	negative	437	0.33
489	none	negative	35	male	outpatient	others	I/II	negative	negative	negative	285	0.30
490	none	negative	24	male	outpatient	sex	I/II	negative	negative	negative	24	0.03
491	none	negative	31	male	outpatient	sex	I/II	negative	negative	negative	113	0.13
492	none	negative	46	male	outpatient	others	I/II	negative	negative	negative	112	0.10
493	none	negative	24	male	outpatient	sex	I/II	negative	negative	negative	859	1.06
494	none	negative	20	male	outpatient	sex	I/II	negative	negative	negative	314	0.59
495	none	negative	23	male	outpatient	sex	I/II	negative	negative	negative	911	1.11
496	none	negative	63	male	outpatient	sex	I/II	negative	negative	negative	339	0.51
497	none	negative	30	male	outpatient	sex	I/II	negative	negative	negative	444	0.54
498	none	negative	34	male	outpatient	others	I/II	negative	negative	negative	609	0.48
499	none	negative	30	male	outpatient	sex	I/II	negative	negative	negative	519	0.62
500	none	negative	65	male	outpatient	sex	I/II	negative	positive	negative	67	0.09
501	none	negative	39	male	outpatient	sex	I/II	negative	positive	negative	438	0.33
502	none	negative	22	male	outpatient	sex	I/II	negative	negative	negative	249	0.35
503	none	negative	31	male	outpatient	sex	I/II	negative	negative	negative	281	0.37
504	none	negative	40	male	outpatient	sex	I/II	negative	negative	negative	324	0.40
505	none	negative	25	male	outpatient	sex	I/II	negative	negative	negative	238	0.41
506	none	negative	64	male	outpatient	sex	I/II	negative	negative	negative	271	0.44
507	none	negative	29	male	outpatient	sex	I/II	negative	negative	negative	410	1.27
508	none	negative	23	male	outpatient	others	I/II	negative	negative	negative	568	0.46
509	none	negative	21	male	outpatient	sex	I/II	negative	negative	negative	565	0.25
510	none	negative	19	female	outpatient	sex	I/II	negative	negative	negative	774	0.50
511	none	negative	21	male	outpatient	sex	I/II	negative	negative	negative	336	0.23
512	none	negative	33	male	outpatient	sex	I/II	negative	negative	negative	226	0.24
513	none	negative	54	female	outpatient	sex	I/II	negative	negative	negative	250	0.22
514	none	negative	25	male	outpatient	others	I/II	negative	negative	negative	376	0.50
515	none	negative	26	male	outpatient	sex	I/II	negative	negative	negative	201	0.36
516	none	negative	30	male	outpatient	sex	I/II	negative	positive	negative	167	0.08
517	none	negative	27	male	outpatient	sex	I/II	negative	negative	negative	783	1.04
518	none	negative	53	male	outpatient	sex	I/II	negative	negative	negative	215	0.28
519	none	negative	20	male	outpatient	sex	I/II	negative	negative	negative	123	0.21
520	none	negative	46	male	outpatient	sex	I/II	negative	negative	negative	480	0.30
521	none	negative	45	female	outpatient	sex	I/II	negative	negative	negative	168	0.37

Guangzhou Eighth People's Hospital
Guangzhou Medical University

522	none	negative	25	male	outpatient	sex	I/II	negative	negative	negative	411	0.38
523	none	negative	42	male	outpatient	others	I/II	negative	negative	negative	324	0.25
524	none	negative	27	male	outpatient	sex	I/II	negative	negative	negative	417	0.32
525	none	negative	29	male	outpatient	sex	I/II	negative	negative	negative	258	0.15
526	none	negative	24	male	outpatient	sex	I/II	negative	negative	negative	407	0.68
527	none	negative	35	male	outpatient	sex	I/II	negative	negative	negative	599	0.36
528	none	negative	25	male	outpatient	sex	I/II	negative	negative	negative	280	0.49
529	none	negative	43	male	outpatient	sex	I/II	negative	negative	negative	364	0.58
530	none	negative	23	male	outpatient	sex	I/II	negative	negative	negative	530	0.34
531	none	negative	29	male	outpatient	sex	I/II	negative	negative	negative	4	0.01
532	none	negative	53	male	outpatient	sex	I/II	negative	negative	negative	558	1.23
533	none	negative	65	male	outpatient	sex	I/II	negative	negative	negative	662	0.82
534	none	negative	27	male	outpatient	sex	I/II	negative	positive	negative	84	0.21
535	none	positive	53	male	outpatient	sex	I/II	negative	negative	negative	8	0.01
536	none	negative	29	male	outpatient	sex	I/II	negative	negative	negative	265	0.42
537	none	negative	29	male	outpatient	sex	I/II	negative	negative	negative	200	0.39
538	none	negative	42	male	outpatient	sex	I/II	negative	negative	negative	337	0.83
539	none	negative	26	male	outpatient	sex	I/II	negative	negative	negative	139	0.28
540	none	negative	62	male	outpatient	sex	I/II	negative	negative	negative	299	0.66
541	none	negative	35	male	outpatient	sex	I/II	negative	negative	negative	338	0.32
542	none	negative	18	male	outpatient	sex	I/II	negative	negative	negative	634	0.82
543	none	negative	22	male	outpatient	sex	I/II	negative	negative	negative	364	0.30
544	none	negative	44	male	outpatient	sex	I/II	negative	negative	negative	706	0.45
545	none	negative	57	male	outpatient	others	I/II	negative	negative	negative	146	0.10
546	none	negative	24	male	outpatient	sex	I/II	negative	negative	negative	602	0.20
547	none	negative	60	male	outpatient	others	I/II	negative	negative	negative	218	0.35
548	none	negative	66	male	outpatient	sex	I/II	negative	positive	negative	150	0.21
549	none	negative	67	male	outpatient	sex	I/II	negative	negative	negative	145	0.36
550	none	negative	23	male	outpatient	sex	I/II	negative	negative	negative	301	0.16
551	none	negative	34	male	outpatient	others	I/II	negative	negative	negative	135	0.17
552	none	negative	53	male	outpatient	sex	I/II	negative	negative	negative	114	0.14
553	none	negative	52	female	outpatient	sex	I/II	negative	negative	negative	32	0.07
554	none	negative	27	male	outpatient	sex	I/II	negative	negative	negative	381	0.23
555	none	negative	45	male	outpatient	others	I/II	negative	negative	negative	438	0.29
556	none	negative	32	male	outpatient	sex	I/II	negative	negative	negative	184	0.37

Guangzhou Eighth People's Hospital
Guangzhou Medical University

557	none	negative	35	male	outpatient	sex	I/II	negative	negative	negative	20	0.03
558	none	negative	63	female	outpatient	sex	I/II	negative	negative	negative	203	0.60
559	none	negative	60	female	outpatient	sex	I/II	negative	negative	negative	382	0.43
560	none	positive	24	male	outpatient	sex	I/II	negative	negative	negative	2	0.02
561	none	negative	66	male	outpatient	sex	I/II	negative	negative	negative	282	0.38
562	none	negative	60	male	outpatient	sex	I/II	negative	negative	negative	108	0.21
563	none	negative	32	male	outpatient	sex	I/II	negative	negative	negative	475	0.25
564	none	negative	24	male	outpatient	sex	I/II	negative	negative	negative	309	0.28
565	none	negative	29	male	outpatient	sex	I/II	negative	negative	negative	123	0.17
566	none	negative	55	male	outpatient	sex	I/II	negative	negative	negative	66	0.25
567	none	negative	74	male	outpatient	sex	I/II	negative	negative	negative	201	0.20
568	none	negative	27	male	outpatient	sex	I/II	negative	negative	negative	272	0.33
569	none	negative	47	male	outpatient	sex	I/II	negative	negative	negative	84	0.39
570	none	negative	28	male	outpatient	others	I/II	negative	negative	negative	289	0.40
571	none	negative	45	male	outpatient	sex	I/II	negative	negative	negative	409	0.48
572	none	negative	20	male	outpatient	sex	I/II	negative	negative	negative	303	0.23
573	none	negative	44	male	outpatient	sex	I/II	negative	negative	negative	33	0.03
574	none	negative	23	male	outpatient	sex	I/II	negative	negative	negative	163	0.13
575	none	negative	33	male	outpatient	others	I/II	negative	negative	negative	427	0.50
576	none	negative	21	male	outpatient	sex	I/II	negative	negative	negative	215	0.38
577	none	negative	61	male	outpatient	sex	I/II	negative	negative	negative	152	0.21
578	none	negative	23	male	outpatient	sex	I/II	negative	negative	negative	188	0.37
579	none	negative	22	male	outpatient	sex	I/II	negative	negative	negative	545	0.40
580	none	negative	21	male	outpatient	sex	I/II	negative	negative	negative	463	0.24
581	none	negative	25	male	outpatient	sex	I/II	negative	negative	negative	131	0.18
582	none	negative	46	male	outpatient	others	I/II	negative	negative	negative	134	0.09
583	none	negative	37	male	outpatient	sex	I/II	negative	negative	negative	65	0.09
584	none	negative	27	male	outpatient	sex	I/II	negative	positive	negative	280	0.44
585	none	negative	46	male	outpatient	sex	I/II	negative	negative	negative	167	0.14
586	none	negative	31	male	outpatient	sex	I/II	negative	negative	negative	103	0.16
587	none	negative	68	male	outpatient	sex	I/II	negative	negative	negative	202	0.43
588	none	negative	22	male	outpatient	sex	I/II	negative	negative	negative	381	1.61
589	none	negative	28	male	outpatient	sex	I/II	negative	negative	negative	228	0.32
590	none	negative	34	male	outpatient	sex	I/II	negative	negative	negative	358	0.55
591	none	negative	28	male	outpatient	sex	I/II	negative	negative	negative	263	0.12

Guangzhou Eighth People's Hospital
Guangzhou Medical University

592	none	negative	32	male	outpatient	sex	I/II	negative	negative	negative	201	0.31
593	none	negative	46	male	outpatient	sex	I/II	negative	positive	negative	435	0.54
594	none	negative	25	male	outpatient	sex	I/II	negative	negative	negative	170	0.27
595	none	negative	36	male	outpatient	sex	I/II	negative	negative	negative	289	0.25
596	none	negative	44	male	outpatient	others	I/II	negative	negative	negative	190	0.15
597	none	negative	26	male	outpatient	sex	I/II	negative	negative	negative	267	0.30
598	none	negative	27	male	outpatient	sex	I/II	negative	negative	negative	385	0.20
599	none	negative	49	male	outpatient	sex	I/II	negative	negative	negative	363	0.61
600	none	negative	26	male	outpatient	sex	I/II	negative	negative	negative	438	0.49
601	none	negative	42	male	outpatient	sex	I/II	negative	negative	positive	295	0.34
602	none	negative	21	male	outpatient	sex	I/II	negative	negative	negative	378	0.54
603	none	negative	36	male	outpatient	sex	I/II	negative	negative	negative	190	0.12
604	none	negative	20	male	outpatient	sex	I/II	negative	negative	negative	192	0.31
605	none	negative	19	male	outpatient	sex	I/II	negative	negative	negative	85	0.13
606	none	negative	41	female	outpatient	sex	I/II	negative	positive	negative	229	0.31
607	none	negative	46	male	outpatient	sex	I/II	negative	negative	negative	106	0.35
608	none	negative	24	male	outpatient	sex	I/II	negative	negative	negative	413	0.61
609	none	negative	33	male	outpatient	sex	I/II	negative	positive	negative	203	0.27
610	none	negative	43	female	outpatient	sex	I/II	negative	negative	negative	189	0.26
611	none	negative	42	male	outpatient	sex	I/II	negative	negative	negative	429	0.18
612	none	negative	50	male	outpatient	sex	I/II	negative	negative	negative	196	0.13
613	none	negative	43	male	outpatient	sex	I/II	negative	negative	negative	189	0.31
614	none	negative	67	male	outpatient	sex	I/II	negative	negative	negative	685	0.90
615	none	negative	25	male	outpatient	sex	I/II	negative	positive	negative	362	0.40
616	none	negative	24	male	outpatient	sex	I/II	negative	negative	negative	332	0.47
617	none	negative	24	male	outpatient	sex	I/II	negative	negative	negative	372	0.39
618	none	negative	36	male	outpatient	sex	I/II	negative	negative	negative	303	0.41
619	none	negative	39	male	outpatient	sex	I/II	negative	negative	negative	139	0.10
620	none	negative	56	female	outpatient	sex	I/II	negative	negative	negative	334	0.52
621	none	negative	51	female	outpatient	others	I/II	negative	negative	negative	321	0.48
622	none	negative	36	male	outpatient	unknown	I/II	negative	negative	negative	513	0.47
623	none	negative	27	female	outpatient	sex	I/II	negative	negative	negative	249	0.31
624	none	negative	27	male	outpatient	others	I/II	negative	negative	negative	457	0.30
625	none	negative	48	male	outpatient	sex	I/II	negative	negative	negative	547	0.83
626	none	negative	47	male	outpatient	blood	I/II	negative	negative	positive	89	0.15

Guangzhou Eighth People's Hospital Guangzhou Medical University

627	none	negative	27	male	outpatient	sex	I/II	negative	positive	negative	321	0.31
628	none	negative	38	male	outpatient	sex	I/II	negative	negative	negative	234	0.26
629	none	negative	39	male	outpatient	sex	I/II	negative	positive	negative	78	0.09
630	none	negative	21	male	outpatient	sex	I/II	negative	negative	negative	281	0.25
631	none	negative	25	male	outpatient	sex	I/II	negative	negative	negative	148	0.16
632	none	negative	33	female	outpatient	sex	I/II	negative	positive	negative	268	0.35
633	none	negative	22	male	outpatient	sex	I/II	negative	negative	negative	192	0.11
634	none	negative	34	female	outpatient	others	I/II	negative	negative	negative	336	0.47
635	none	negative	21	male	outpatient	sex	I/II	negative	negative	negative	192	0.11
636	none	negative	27	male	outpatient	sex	I/II	negative	negative	negative	194	0.17
637	none	negative	46	male	outpatient	sex	I/II	negative	positive	negative	201	0.39
638	none	negative	22	male	outpatient	sex	I/II	negative	negative	negative	537	0.44
639	none	negative	46	male	outpatient	sex	I/II	negative	positive	negative	20	0.04
640	none	negative	29	male	outpatient	others	I/II	negative	negative	negative	368	0.39
641	none	negative	57	male	outpatient	sex	I/II	negative	negative	negative	151	0.35
642	none	negative	52	male	outpatient	sex	I/II	negative	negative	negative	259	0.30
643	none	negative	52	female	outpatient	sex	I/II	negative	negative	negative	149	0.39
644	none	negative	36	male	outpatient	sex	I/II	negative	negative	negative	488	0.45
645	none	negative	32	male	outpatient	sex	I/II	negative	negative	negative	189	0.14
646	none	negative	18	male	outpatient	sex	I/II	negative	negative	negative	354	0.49
647	none	negative	30	male	outpatient	sex	I/II	negative	negative	negative	280	0.39
648	none	negative	32	female	outpatient	blood	I/II	negative	negative	negative	266	0.35
649	none	negative	47	male	outpatient	sex	I/II	negative	negative	negative	843	0.94
650	none	negative	46	male	outpatient	sex	I/II	negative	negative	negative	154	0.17
651	none	negative	32	male	outpatient	sex	I/II	negative	negative	negative	114	0.22
652	none	negative	38	male	outpatient	sex	I/II	negative	negative	negative	213	0.16
653	none	negative	53	male	outpatient	sex	I/II	negative	negative	negative	347	0.46
654	none	negative	23	male	outpatient	sex	I/II	negative	negative	negative	454	0.35
655	none	negative	35	male	outpatient	sex	I/II	negative	negative	negative	483	0.32
656	none	negative	30	male	outpatient	sex	I/II	negative	negative	negative	374	0.21
657	none	negative	22	male	outpatient	sex	I/II	negative	negative	negative	399	0.40
658	none	negative	51	female	outpatient	sex	I/II	negative	negative	negative	195	0.20
659	none	negative	70	male	outpatient	others	I/II	negative	negative	negative	197	0.27
660	none	negative	56	female	outpatient	sex	I/II	negative	negative	negative	417	1.13
661	none	negative	33	male	outpatient	sex	I/II	negative	negative	negative	273	0.29

Guangzhou Eighth People's Hospital
Guangzhou Medical University

662	none	negative	21	male	outpatient	sex	I/II	negative	negative	negative	450	0.26
663	none	negative	26	male	outpatient	sex	I/II	negative	negative	negative	204	0.42
664	none	negative	21	male	outpatient	sex	I/II	negative	negative	negative	203	0.40
665	none	negative	48	female	outpatient	sex	I/II	negative	negative	negative	483	0.30
666	none	negative	39	male	outpatient	sex	I/II	negative	negative	negative	225	0.14
667	none	negative	36	male	outpatient	sex	I/II	negative	positive	negative	183	0.15
668	none	negative	23	male	outpatient	sex	I/II	negative	negative	negative	379	0.36
669	none	negative	35	male	outpatient	sex	I/II	negative	negative	negative	252	0.52
670	none	negative	33	male	outpatient	unknown	I/II	negative	negative	negative	217	0.20
671	none	negative	43	female	outpatient	sex	I/II	negative	negative	negative	132	0.27
672	none	negative	25	male	outpatient	sex	I/II	negative	negative	negative	323	0.34
673	none	negative	25	male	outpatient	sex	I/II	negative	negative	negative	509	0.39
674	none	negative	42	male	outpatient	sex	I/II	negative	negative	negative	445	0.45
675	none	negative	47	female	outpatient	sex	I/II	negative	negative	negative	423	0.64
676	none	negative	37	male	outpatient	sex	I/II	negative	negative	negative	272	0.25
677	none	negative	47	male	outpatient	sex	I/II	negative	negative	negative	74	0.19
678	none	negative	41	male	outpatient	sex	I/II	negative	negative	negative	286	0.34
679	none	negative	48	male	outpatient	sex	I/II	negative	negative	negative	6	0.03
680	none	negative	54	female	outpatient	sex	I/II	negative	negative	negative	135	0.14
681	none	negative	27	male	outpatient	sex	I/II	negative	negative	negative	252	0.39
682	none	negative	27	male	outpatient	sex	I/II	negative	negative	negative	323	0.39
683	none	negative	37	male	outpatient	sex	I/II	negative	negative	negative	53	0.17
684	none	negative	27	male	outpatient	sex	I/II	negative	negative	negative	282	0.47
685	none	negative	27	female	outpatient	sex	I/II	negative	negative	negative	163	0.27
686	none	negative	54	female	outpatient	sex	I/II	negative	negative	negative	65	0.28
687	none	negative	21	male	outpatient	sex	I/II	negative	positive	negative	414	0.38
688	none	negative	42	male	outpatient	sex	I/II	negative	negative	negative	290	0.27
689	none	negative	26	female	outpatient	sex	I/II	negative	positive	negative	224	0.37
690	none	negative	23	female	outpatient	sex	I/II	negative	negative	negative	277	0.40
691	none	negative	22	male	outpatient	sex	I/II	negative	negative	negative	185	0.19
692	none	negative	34	male	outpatient	sex	I/II	negative	positive	negative	116	0.18
693	none	negative	27	male	outpatient	sex	I/II	negative	negative	negative	296	0.44
694	none	negative	25	male	outpatient	sex	I/II	negative	negative	negative	270	0.39
695	none	negative	34	male	outpatient	sex	I/II	negative	negative	negative	286	0.52
696	none	negative	37	male	outpatient	unknown	I/II	negative	negative	negative	297	0.52

Guangzhou Eighth People's Hospital
Guangzhou Medical University

697	none	positive	23	male	outpatient	sex	I/II	negative	negative	negative	148	0.23
698	none	negative	53	male	outpatient	others	I/II	negative	negative	negative	410	0.27
699	none	negative	38	female	outpatient	sex	I/II	negative	negative	negative	277	0.42
700	none	negative	25	male	outpatient	sex	I/II	negative	negative	negative	570	0.47
701	none	negative	46	male	outpatient	sex	I/II	negative	negative	negative	241	0.26
702	none	negative	48	female	outpatient	sex	I/II	negative	negative	negative	185	0.28
703	none	negative	33	male	outpatient	sex	I/II	negative	negative	negative	402	0.34
704	none	negative	39	male	outpatient	sex	I/II	negative	positive	negative	627	0.68
705	none	negative	20	male	outpatient	sex	I/II	negative	negative	negative	287	0.66
706	none	negative	37	male	outpatient	sex	I/II	negative	negative	negative	176	0.16
707	none	negative	29	male	outpatient	sex	I/II	negative	positive	negative	259	0.37
708	none	negative	50	female	outpatient	sex	I/II	negative	negative	negative	322	0.56
709	none	negative	32	female	outpatient	others	I/II	negative	negative	negative	38	0.05
710	none	negative	42	male	outpatient	sex	I/II	negative	negative	negative	69	0.14
711	none	negative	49	male	outpatient	sex	I/II	negative	negative	negative	365	0.98
712	none	negative	45	male	outpatient	sex	I/II	negative	negative	negative	182	0.26
713	none	negative	27	male	outpatient	unknown	I/II	negative	negative	negative	590	0.55
714	none	negative	37	male	outpatient	sex	I/II	negative	negative	negative	141	0.08
715	none	negative	56	male	outpatient	sex	I/II	negative	negative	negative	447	0.38
716	none	negative	70	male	outpatient	sex	I/II	negative	negative	negative	169	0.12
717	none	negative	34	male	outpatient	sex	I/II	negative	negative	negative	50	0.09
718	none	negative	42	male	outpatient	others	I/II	negative	negative	negative	125	0.14
719	none	negative	27	male	outpatient	sex	I/II	negative	negative	negative	147	0.14
720	none	negative	50	male	outpatient	sex	I/II	negative	negative	negative	35	0.08
721	none	negative	36	male	outpatient	sex	I/II	negative	negative	negative	32	0.19
722	none	negative	27	male	outpatient	sex	I/II	negative	negative	negative	164	0.12
723	none	negative	28	male	outpatient	sex	I/II	negative	negative	negative	189	0.10
724	none	negative	29	male	outpatient	sex	I/II	negative	negative	negative	128	0.23
725	none	negative	21	male	outpatient	unknown	I/II	negative	positive	negative	89	0.24
726	none	negative	25	male	outpatient	others	I/II	negative	positive	negative	22	0.03
727	none	negative	26	male	outpatient	sex	I/II	negative	negative	negative	124	0.13
728	none	negative	26	male	outpatient	sex	I/II	negative	negative	negative	106	0.14
729	none	negative	32	male	outpatient	sex	I/II	negative	negative	negative	162	0.18
730	none	negative	66	male	outpatient	sex	I/II	negative	positive	negative	122	0.22
731	none	negative	25	male	outpatient	sex	I/II	negative	negative	negative	36	0.04

Guangzhou Eighth People's Hospital
Guangzhou Medical University

732	none	negative	25	male	outpatient	sex	I/II	negative	negative	negative	185	0.20
733	none	negative	50	male	outpatient	sex	III	positive	negative	negative	38	0.09
734	none	negative	43	male	outpatient	sex	I/II	negative	positive	negative	86	0.09
735	none	negative	28	male	outpatient	sex	I/II	negative	negative	negative	59	0.07
736	none	negative	42	female	outpatient	sex	I/II	negative	negative	negative	137	0.32
737	none	negative	25	female	outpatient	sex	I/II	negative	negative	negative	152	0.25
738	none	negative	27	male	outpatient	sex	I/II	negative	negative	negative	147	0.16
739	none	positive	35	male	outpatient	sex	I/II	negative	negative	negative	1	0.02
740	none	negative	46	male	outpatient	sex	I/II	negative	negative	negative	134	0.17
741	none	negative	32	male	outpatient	sex	I/II	negative	negative	negative	118	0.05
742	none	negative	71	male	outpatient	sex	I/II	negative	negative	negative	152	0.38
743	none	negative	70	female	outpatient	sex	I/II	negative	negative	negative	137	0.22
744	none	positive	29	male	outpatient	sex	I/II	negative	negative	negative	144	0.14
745	none	negative	27	male	outpatient	sex	I/II	negative	negative	negative	158	0.16
746	none	negative	33	male	outpatient	sex	I/II	negative	positive	negative	128	0.17
747	none	negative	24	male	outpatient	sex	I/II	negative	negative	negative	58	0.07
748	none	negative	20	male	outpatient	sex	I/II	negative	negative	negative	143	0.18
749	none	negative	59	male	outpatient	sex	I/II	negative	negative	negative	168	0.63
750	none	negative	32	male	outpatient	sex	I/II	negative	negative	negative	29	0.05
751	none	positive	61	male	outpatient	sex	I/II	negative	negative	negative	24	0.04
752	none	negative	37	female	outpatient	sex	I/II	negative	negative	negative	77	0.22
753	none	negative	60	male	outpatient	sex	I/II	negative	negative	negative	178	0.37
754	none	negative	37	male	outpatient	sex	I/II	negative	negative	negative	82	0.06
755	none	negative	35	male	outpatient	sex	I/II	negative	negative	negative	169	0.17
756	none	negative	50	male	outpatient	sex	I/II	negative	negative	negative	126	0.31
757	none	negative	24	male	outpatient	sex	I/II	negative	negative	negative	180	0.27
758	none	negative	53	male	outpatient	sex	I/II	negative	positive	negative	189	0.28
759	none	negative	37	male	outpatient	sex	I/II	negative	negative	negative	126	0.19
760	none	negative	29	male	outpatient	sex	I/II	negative	negative	negative	143	0.32
761	none	negative	19	male	outpatient	sex	I/II	negative	negative	negative	123	0.25
762	none	negative	60	male	outpatient	sex	I/II	negative	negative	negative	94	0.19
763	none	negative	26	male	outpatient	sex	I/II	negative	negative	negative	188	0.04
764	none	negative	33	male	outpatient	sex	I/II	negative	negative	negative	116	0.14
765	none	negative	44	male	outpatient	sex	I/II	negative	negative	negative	196	0.24
766	none	negative	38	male	outpatient	sex	I/II	negative	negative	negative	168	0.18

Guangzhou Eighth People's Hospital
Guangzhou Medical University

767	none	negative	35	male	outpatient	sex	I/II	negative	positive	negative	19	0.03
768	none	negative	62	male	outpatient	sex	I/II	negative	negative	negative	103	0.15
769	none	negative	45	male	outpatient	others	I/II	negative	negative	negative	47	0.10
770	none	negative	52	male	outpatient	blood	I/II	negative	positive	positive	61	0.06
771	none	negative	33	male	outpatient	sex	I/II	negative	negative	negative	189	0.14
772	none	negative	48	female	outpatient	sex	I/II	negative	negative	negative	142	0.15
773	none	negative	30	female	outpatient	sex	I/II	negative	negative	negative	145	0.09
774	none	negative	35	male	outpatient	sex	I/II	negative	negative	negative	137	0.21
775	none	negative	24	male	outpatient	sex	I/II	negative	negative	negative	113	0.16
776	none	negative	31	male	outpatient	sex	I/II	negative	negative	negative	156	0.11
777	none	negative	27	male	outpatient	sex	I/II	negative	negative	negative	68	0.09
778	none	negative	21	female	outpatient	sex	I/II	negative	negative	negative	194	0.37
779	none	negative	28	male	outpatient	sex	I/II	negative	negative	negative	147	0.18
780	none	negative	66	male	outpatient	sex	I/II	negative	negative	negative	182	0.07
781	none	negative	44	male	outpatient	sex	I/II	negative	negative	negative	114	0.09
782	none	negative	24	male	outpatient	sex	I/II	negative	negative	negative	183	0.15
783	none	negative	60	male	outpatient	sex	I/II	negative	negative	negative	105	0.09
784	none	negative	23	male	outpatient	sex	I/II	negative	positive	negative	269	0.43

Guangzhou Eighth People's Hospital
Guangzhou Medical University
