# Supplementary material for: An evaluation of Mp1p antigen screening for talaromycosis in HIV-infected antiretroviral therapy-naïve population in Guangdong, China
Source: PLoS Negl Trop Dis. 2023 Nov 27;17(11):e0011785. doi: 10.1371/journal.pntd.0011785 (PMC10703259; doi:10.1371/journal.pntd.0011785)
Supplement: Attachment — Submitted filename: Response to reviewers.docx [file pntd.0011785.s002.docx]

Re: (Manuscript ID) PNTD-D-23-00914

An evaluation of Mp1p antigen screening for talaromycosis in HIV-infected antiretroviral therapy-naïve population in Guangdong, China.

**Reviewer's Responses to Questions**

**Key Review Criteria Required for Acceptance?**

As you describe the new analyses required for acceptance, please consider the following:

**Methods**

-Are the objectives of the study clearly articulated with a clear testable hypothesis stated?

-Is the study design appropriate to address the stated objectives?

-Is the population clearly described and appropriate for the hypothesis being tested?

-Is the sample size sufficient to ensure adequate power to address the hypothesis being tested?

-Were correct statistical analysis used to support conclusions?

-Are there concerns about ethical or regulatory requirements being met?

**Reviewer #1**: No, the objectives of the study weren't clearly articulated with a clear testable hypothesis stated.

Yes, the study design was appropriate to address the stated objectives.

Yes, the population was clearly described and appropriate for the hypothesis being tested.

Yes, the sample size was sufficient to ensure adequate power to address the hypothesis being tested.

Yes, statistical analysis was correct used to support conclusions.

No, there aren't concerns about ethical or regulatory requirements being met.

**Response**: Thanks for your positive attitude and constructive comments. We have modified the introduction of this study and focused the objective on the assessment of the utility of Mp1p antigen screening among HIV-infected ART-naïve population.

**Reviewer #2**: The methods for screening patients with Mp1p antigen testing followed by culture testing are sufficient to investigate the efficacy of the antigen test and support the conclusions in the manuscript. Ultimately, these data will benefit from a larger sample size, but the sample size in the present study appears sufficient to support the claims.

**Response**: We really appreciate your positive attitude and constructive comments.

--------------------

**Results**

-Does the analysis presented match the analysis plan?

-Are the results clearly and completely presented?

-Are the figures (Tables, Images) of sufficient quality for clarity?

**Reviewer #1**: Yes, the analysis presented match the analysis plan.

Yes, the results were clearly and completely presented.

No, the figures weren't of sufficient quality for clarity.

Yes, the Tables were of sufficient quality for clarity.

There is not any pictures.

**Response**: Thanks for your kind suggestions. The figures have been rebuilt to improve their quality and to meet the figure file requirements.

**Reviewer #2**: The results of the study are clear, if not ground-breaking. The authors show that Mp1p antigen screening is effective for detection in *Talaromycosis* in HIV/AIDS patients in Southern China. In many cases, the text could be written in a way that would more clearly convey the findings, as it is often unclear which association the researchers are attempting to make. For example, the text in lines 233-241 could be interpreted as Talaromycosis causing decreased CD4 counts in patients, rather than low CD4 counts leaving patients susceptible to Talaromycosis. This should be clarified throughout the text.

**Response:** Thanks for your constructive comments, we have adjusted the description of the results, writing in the way that convey the finding more accurately.

--------------------

**Conclusions**

-Are the conclusions supported by the data presented?

-Are the limitations of analysis clearly described?

-Do the authors discuss how these data can be helpful to advance our understanding of the topic under study?

-Is public health relevance addressed?

**Reviewer #1**: No, the conclusions weren't supported by the data presented.

No, the limitations of analysis weren't clearly described.

Yes, the authors discussed how these data can be helpful to advance our understanding of the topic under study.

Yes, public health was relevance addressed.

**Response**: Thanks for your critical reminder. We have improved the conclusion to make it more logical and to be more closely supported by the results. Meanwhile, the limitations of the study were restructured, with accordingly explanations and possibilities of further studies.

**Reviewer #2**: The basic conclusion of the paper is that Mp1p antigen screening can be an effective tool for more efficient diagnosis of Talaromycosis, especially in HIV/AIDS patients with low CD4 counts. The data suggest this is a sound conclusion. Further discussion of the benefits of antigen testing would be useful. For example, how cost effective is this antigen test? How much time does it save, practically, compared to culture testing? Some data to this effect would bolster the authors' claims that antigen testing would be an effective strategy to implement earlier antifungal treatment in patients with low CD4 counts.

**Response:** Thanks for your positive attitude. We have stressed the advantage of time-saving, less expensive and avoiding invasive methods to obtain sample of Mp1p antigen test.

--------------------

**Editorial and Data Presentation Modifications?**

Use this section for editorial suggestions as well as relatively minor modifications of existing data that would enhance clarity. If the only modifications needed are minor and/or editorial, you may wish to recommend “Minor Revision” or “Accept”.

**Reviewer #1**: ● The aim was clear.

● Abstract (291 words) was clear what the study found and how they did it

● The title “An evaluation of Mp1p antigen screening for talaromycosis in HIV-infected antiretroviral therapy-naïve population in Guangdong, China” was informative and relevant.

● Author Summary was clear and understandable, and the author summarized all the things that should be stated.

●The references were: 26 references (17 new and 9 old references).

● The references must come from current scientific journals (c. 80% published in the last 10 years)

● Referenced were correctly.

● Appropriate were key studies included.

**Response**: We have changed the number of references to 25, including 21 references published in the last 10 years.

**Reviewer #2**: Editing for grammar throughout the manuscript will be crucial.

Terms like PPV, NPV, kappa, and OI should be defined where they are first used.

In the introduction, the authors state that the mortality rate of T. marneffei in HIV patients is ~33%, a figure which comes from a small study by Son et al in 2014, but it seems more recent estimates, as in Jiang 2019 (Clinical Microbiology and Infection) may be more accurate.

Line 164 “The cut-off value was 0.1” needs units.

**Response**:

1. The grammar of the manuscript has been edited.

2. The full name or the explanations of the listed terms have been added where they are first used.

3. A more accurate data from a more recent reference has been cited here.

4. 0.1 is the value of absorbance, and absorbance is a dimensionless quantity. Therefore, there is not a units for 0.1.

--------------------

**Summary and General Comments**

Use this section to provide overall comments, discuss strengths/weaknesses of the study, novelty, significance, general execution and scholarship. You may also include additional comments for the author, including concerns about dual publication, research ethics, or publication ethics. If requesting major revision, please articulate the new experiments that are needed.

**Reviewer #1**:

Introduction / background

●Introduction wasn’t clear what is known about this topic, please add one page more about the topic with new references.

● The research questions were clearly outlined.

● The research questions were justified given what is known about the topic.

**Response**: Four references published after 2016 have been cited in the new manuscript, substituting their counterparts in the former manuscript, to introduce the background information of the study more clearly.

Methods: Experimental:

● The process of subject was selection clear.

● The variables were defined and measured appropriately.

● The study methods were valid and reliable.

● The sample size was very suitable for such a study.

● Great job from the authors for having an Ethics statement.

● There were enough detail in order to replicate the study.

● There weren't enough details on the method of Specimen culture for talaromycosis.

**Response**: This study aimed to explore a novel method to screen talaromycosis, so we would like to provide more detailed information of Mp1p antigen test. What’s more, specimen culture has been conducted for many years and the standard culture techniques have been reported in multiple essays and guidelines, so we prefer to cite the previous references to show this method for the purpose of brevity and avoiding redundancy.

Results:

● The data was presented in an appropriate way.

● Tables were relevant and clearly presented.

●Figures weren’t relevant and clearly presented.

● The units, rounding, and number of decimals were appropriate.

● Titles, columns, and rows were labelled correctly and clearly.

● Categories were grouped appropriately.

● The text in the results was added to the data.

● I’m clear about what is a statistically significant result.

● I’m clear about what is a practically meaningful result.

●I didn't see any pictures that were crisp and beautifully done.

**Response**: The figures have been rebuilt to improve their quality and to meet the figure file requirements.

Discussion and Conclusions:

● The results were discussed from multiple angles and placed into context without being overinterpreted.

●The conclusions weren’t to answer the aims of the study.

● The conclusions weren't supported by references or results.

● The limitations of the study give opportunities to make future research.

**Response**: We have focused the objective of the study on the assessment of the utility of Mp1p antigen screening among HIV-infected ART-naïve population. The conclusion also be improved to make it more logical and be more closely supported by the results. Meanwhile, six references published recently were cited to support the aim of the study.

**Reviewer #2**: This study makes a reasonable case that implementation of Mp1p antigen testing is a sensitive and reliable assay for Talaromycosis in HIV/AIDS patients and could be used for early screening in endemic areas to get patients antifungal treatment more efficiently. These data are potentially informative and useful for this specific niche. Ultimately, the conclusions would benefit from additional data or discussion of the practicality of implementing this screening test.

**Response**: We really appreciate your positive attitude for our study.
